# Mitigating Oxidative Stress and Promoting Cellular Longevity with Mushroom Extracts

**DOI:** 10.3390/foods13244028

**Published:** 2024-12-13

**Authors:** Menna-Allah E. Abdelkader, Hatungimana Mediatrice, Dongmei Lin, Zhanxi Lin, Sarah A. Aggag

**Affiliations:** 1 China National Engineering Research Center of Juncao Technology, College of Life Sciences, Fujian Agriculture and Forestry University, Fuzhou 350002, China; menna.elsayed@alexu.edu.eg (M.-A.E.A.); mediatunga@gmail.com (H.M.); lzxjuncao@163.com (Z.L.); 2 Department of Genetics, Faculty of Agriculture, Alexandria University, Alexandria 21545, Egypt; sarah.aggag@alexu.edu.eg; 3 Rwanda Agriculture and Animal Resources Development Board, P.O. Box 5016 Kigali, Rwanda

**Keywords:** mushroom, D-gal, anti-aging, antioxidants, cell cycle, DNA damage, ROS, telomere length, IL-genes

## Abstract

Oxidative stress can disrupt the body’s ability to fight harmful free radicals, leading to premature aging and various health complications. This study investigated the antioxidant and anti-aging properties of four medicinal and edible mushrooms: *Ganoderma lucidum*, *Hericium erinaceus*, *Pleurotus ostreatus*, and *Agaricus bisporus*. The antioxidant activity of mushroom extracts was evaluated using (DPPH-ABTS-Reducing power). The anti-aging effects were assessed using Human Skin Fibroblasts (HSF) cells subjected to D-galactose-induced aging (30 g/L/72 h) and treated with mushroom extracts (0.03–0.25 mg/mL/72 h). The results demonstrated that all mushrooms have significant antioxidant and anti-aging properties, with low concentrations of extracts (0.03 mg/mL) effectively promoting cell proliferation at an 87% rate in the *Agaricus bisporus* extract, enhancing cell cycle progression by reducing the arrested cells in the G0/G1 phase to 75%, and promoting DNA synthesis in S phase by more than 16.36% in the *Hericium erinaceus* extract. Additionally, the extracts reduced DNA damage and Reactive Oxygen Species (ROS) levels, protecting cells from oxidative stress and potentially contributing to anti-aging effects. The mushrooms also exhibited immunomodulatory and anti-inflammatory effects by upregulating the IL-2, IL-4, and downregulating IL-6 expression, indicating their potential to promote general health. These findings suggest the potential of mushroom extracts as natural agents for reducing the negative effects of aging while promoting cellular health. Further research is required to explore the specific bioactive compounds responsible for these beneficial effects and to evaluate their efficacy in vivo.

## 1. Introduction

Prized for centuries as both food and medicine, mushrooms have captured the attention of modern science. These fungal wonders are packed with potent compounds, including antioxidants, polysaccharides, triterpenoids, and potential antiviral and anti-tumorigenic agents. Ancient cultures, such as the Chinese and Egyptians, recognized their therapeutic potential, incorporating them into royal diets [1]. Mushrooms can boost endogenous antioxidants and enzymes, preventing diseases like hypertension, cancer, and AIDS. Recent molecular research has discovered bioactive compounds in mushrooms, including polysaccharides that improve the immune system and eliminate hyperoxide radicals, contributing to aging. Additionally, their capacity to grow on agricultural waste and offer a long-term waste management solution makes them an important resource for sustainable development [2].

Scientific studies show that mushrooms have pharmacological potential, particularly *Ganoderma lucidum*, *Lentinula edodes*, and *Trametes versicolor*. These mushrooms are known for their immunostimulant properties and are used in complementary cancer therapy [3,4]. *Ganoderma lucidum* extract has been shown to eliminate hyperoxide radicals, contributing to anti-aging effects [5]. Likewise, *Hericium erinaceus* has been found to have active compounds that delay neuronal cell death in rats with neurodegenerative diseases, as well as antidepressant and anxiolytic potential [6,7]. *Pleurotus ostreatus*, the most common mushroom grown worldwide, provides essential nutrients and bioactive substances [8,9]. It has numerous pharmacological actions and medicinal properties, including anti-inflammatory, immunomodulatory, antiviral, antidiabetic, tumor chemopreventive, and prebiotic properties [10,11]. It can also reduce the risk of age-related diseases linked to free radical involvement and enhance antioxidant levels during aging. *Agaricus bisporus*, also known as the white button mushroom, is a widely available edible mushroom [12] with numerous nutrients and bioactive substances [13,14]. It has potential as an antioxidant, anticancer, anti-inflammatory, and anti-obesity agent [15,16]. It can prevent the effects of aging and neurodegenerative diseases [17,18].

Antioxidants are crucial for human health [19], and there is a growing global movement towards using natural antioxidants from dietary vegetables or medicinal plants due to several problems related to synthetic drugs, leading to an increasing demand for nutritional supplements [4]. Oxidative stress is globally known as an imbalance between oxidizing agents and antioxidant defenses in favor of the oxidants. This imbalance can disrupt redox signaling pathways, control, and/or cause molecular impairment.

This imbalance may arise from a deficiency in antioxidants like glutathione, superoxide dismutase, catalase, and vitamin E, or an excess of oxidants (reactive species) such as free radicals (superoxide anion, hydroxyl, and nitric oxide) and non-radical molecules (hydrogen peroxide, hypochlorous acid) [20]. However, oxidative stress is a double-edged sword, capable of both beneficial (eustress) and harmful (distress) effects. Oxidative eustress refers to physiological oxidative stress, which is normally involved in various cellular processes and contributes to overall health. Conversely, oxidative distress or supraphysiological oxidative stress can overwhelm the body’s antioxidant defenses, resulting in oxidative damage and contributing to various diseases [21]. Depending on the cellular environment, oxidative stress can cause cellular and molecular dysfunction, ultimately contributing to aging, metabolic disorders, and chronic diseases, including aging-related diseases, diabetes, cardiovascular, and autoimmune disorders, infections, cancer, arthritis, cataracts, neurological conditions, multiple sclerosis, and long-term neurodegenerative diseases like Alzheimer’s, Parkinson’s, and Huntington’s [2,22]. Oxidative stress accelerates senescence-based telomere loss, whereas antioxidants can slow it down. Oxidative damage is not as well repaired in telomeric DNA as it is in other parts of the chromosome [23].

Most studies focus on the nutritional benefits and medicinal importance of edible mushrooms, with limited knowledge of their antioxidant activity and potential to prevent of oxidative stress in aging individuals. *Agaricus bisporus* and *Pleurotus ostreatus* are the most popular edible farmed mushrooms. They are widely distributed and present in both temperate and tropical regions of the planet. Because of their medicinal and culinary properties and high nutritional value, they are regarded as two of the most significant mushrooms [14,24]. *Ganoderma lucidum* usually termed Reishi, is a medicinal fungus with a history of over 2000 years of therapeutic use in Asia; it is the most popular medicinal fungus in China and is also known as the mushroom of immortality [25]. *Hericium erinaceus*, popularly known as lion’s mane mushroom, is a medicinal and edible fungus that, for more than two millennia, was prized for its mouthwatering flavor, nutritious content, and therapeutic properties [26].

The present study aims to compare the nutritional and medicinal benefits of four edible and medicinal mushrooms by investigating their antioxidant potential. The study also evaluated the impact of mushrooms on cell viability, cell morphology, cell cycle, and DNA damage. Additionally, the study assessed the gene expression of telomeres and genes associated with immunomodulators and pro-inflammatory cytokines.

## 2. Materials and Methods

### 2.1. Sample Collection

Four types of mushrooms were used, as follows: Fresh edible mushrooms (*Agaricus bisporus* (*AB*), and *Pleurotus ostreatus* (*PO*)) were collected from the Agriculture Research Centre, Alexandria, Egypt (ARC) in October 2021 and transferred for processing on the day of harvest. The medicinal mushrooms (*Ganoderma lucidum* (*GL*), *Hericium erinaceus* (*HE*)) were purchased from DXN Company, Malaysia.

### 2.2. Preparation of an Aqueous Mushroom Extracts

The fresh mushrooms were chopped into thin slices, lyophilized (Labocon, Hampshire, UK), and ground into a fine powder. Four grams of powdered mushroom sample were added to 100 mL of hot sterile distlled water (80 °C) for 3 h with stirring (Gallenkamp, Cambridge, UK) and then incubated overnight at 25 °C (Heraeus, Hanau, Germany) [27]. The extracts were kept in small aliquots and stored at −20 °C (Zanussi, Pordenone, Italy). 

### 2.3. Determination of Antioxidant Potential

The antioxidant potential of four different mushroom extracts was measured using various methods, including α, α-diphenyl-β-picrylhydrazyl (DPPH), reducing power, and 2,2′-azino-bis (3-ethylbenzothiazoline-6-sulfonic acid) (ABTS). The antioxidant activities were evaluated compared to ascorbic acid (AscH) as a standard antioxidant. The formula used to calculate the IC_50_ value (50% inhibitory concentration) for each radical is [Abs Control − Abs Sample/Abs Control × 100] at various mushroom extract concentrations when compared with a standard antioxidant [28]. Different concentrations (0.005–0.2 mg/mL) of each studied sample and AscH were prepared for each experiment. Three test tubes were prepared separately for each concentration. The free radical scavenging activity was measured by the DPPH method as proposed by Brand-Williams et al. [29], with some modifications. The mixture of DPPH^•^ and the studied samples, or AscH, were incubated for 30 min in the dark at room temperature, and then the absorbance of the non-scavenged radical was measured at 517 nm using a spectrophotometer. The method of the ABTS^+•^ scavenging assay was performed based on Abdel-Razek et al. [30]. A fresh ABTS^+•^ working solution was prepared for each assay by mixing ABTS and potassium persulfate solutions. The inhibitory concentrations at which 50% (IC_50_) (mg/mL) of DPPH^•^ and ABTS^+•^ are scavenged were reported. The different mushroom extracts reacted with ABTS^+•^, and the absorbance was measured at 734 nm using the spectrophotometer. The reducing power of the mushroom extracts was determined according to the slightly modified ferric ion-reducing antioxidant power assay (FRAP) [31]. The mushroom extract and AscH (standard) were mixed with sodium phosphate buffer and 1% potassium ferricyanide. The mixtures were incubated at 50 °C for 20 min. Afterward, each mixture was acidified with 10% trichloroacetic acid and centrifuged at 2795× *g* for 10 min. The upper layer was mixed with an equal volume of deionized water and 0.1% ferric chloride and allowed to stand for 10 min to produce a blue color solution with a maximum absorbance at 700 nm. The reducing power was reported as EC_50_ (mg/mL), an effective concentration, at which the absorbance is 0.5.

### 2.4. Evaluation of the Impact on Aging Caused by D-Galactose in HSF Cells

The Human Skin Fibroblasts (HSF) cell line was purchased from the National Cancer Institute (NCI), Cairo University, Cairo, Egypt. HSF cells were cultured in DMEM medium containing 10% fetal bovine serum (FBS) and 1% penicillin/streptomycin (Cegrogen Biotech, Stadtallendorf, Germany) and incubated in a humidified incubator with 5% CO_2_ at 37 °C.

To establish the HSF aging model, the cells were exposed to 30 g/L D-galactose (300 mg + 100 µL DMSO) for 72 h [32]; this concentration and duration of exposure cause aging in the cell according to previous research, where D-gal causes oxidative stress and decreases cell viability. This leads to an increase in senescenct cells, telomere shortening, and accumulation of DNA damage.

#### Cell Viability Determination

Cell viability was determined by the MTT (3-(4,5-dimethylthiazol-2-yl)-2,5-diphenyltetrazolium bromide) assay (Invitrogen, Thermo Fisher Scientific Inc., Carlsbad, CA, USA) [33]. A total of 100 µL of normal and aging HSF cells (7 × 10^3^ cells/well) were added to 96-well plates and incubated for 24 h. Subsequently, the cells were treated with 100 µL/well of different mushroom extracts (*AB*, *PO*, *GL*, and *HE*) with various concentrations (0.50, 0.25, 0.10, 0.06, 0.03, and 0.01 mg/mL) and then incubated in a 37 °C, 5% CO_2_ humidified incubator for 48 and 72 h. Absorbance was measured at 490 nm using a microplate reader (Bio-Rad Benchmark, Tokyo, Japan). The cell viability was calculated as the ratio of absorbance values as follows:

Viability% = Av Sample-Blank/Av Control-Blank × 100.

The concentration required for 50% cell viability (IC_50_) was determined by plotting a dose-response curve.

Based on the MTT assay results, mushroom extracts at concentrations of 0.25 and 0.03 mg/mL were found to be optimal for enhancing cell viability and reducing the aging effects of D-galactose after 72 h. The low concentration (0.03 mg/mL) refers to the lowest dose at which the mushroom extracts showed the best cell viability (~≥90%), while the high concentration (0.25 mg/mL) refers to the highest dose at which the extracts maintained good cell viability (>50%). 

### 2.5. Aging HSF Cells Induced by D-Gal and Treatment

**Figure 1:**
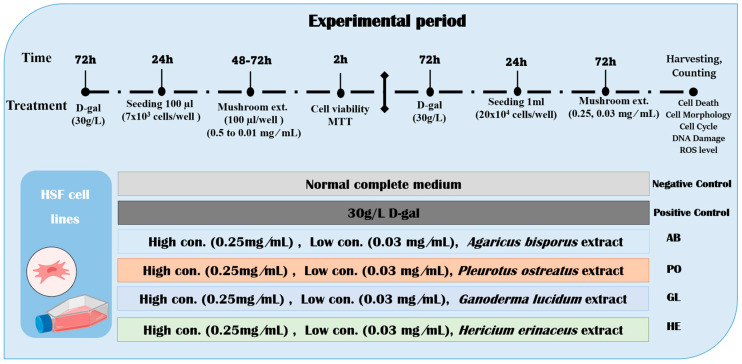
Illustration of the timeline of the cell line experiment. The HSF cells were exposed to 30 g/L D-gal for 72 h to induce aging, then treated with four species of mushroom extracts (0.50 to 0.01 mg/mL) for 48 and 72 h. MTT was used to determine the cell viability after 48 and 72 h. After that, 0.25 and 0.03 mg/mL were used as high and low concentrations of mushroom extracts for a 72 h experiment period. After cell harvesting, cell death, cell morphology, cell cycle, DNA damage, ROS level, and gene expression were determined.

When the cells reached 80% confluency, the HSF of normal and aging cells were seeded into 6-well plates at 10 × 10^4^ cells/well with cover glass for cell morphology analysis and at 20 × 10^4^ cells/well for the other analysis. After 24 h, the aging cells were treated with different mushroom extracts (AB, PO, GL, and HE) at 0.25, and 0.03 mg/mL and then incubated for 72 h in a 37 °C, 5% CO_2_ humidified incubator. The cells were harvested by centrifugation at 500× *g* for 10 min, and the pellet was re-suspended in PBS. All analyses were performed in triplicate (Figure 1). 

#### 2.5.1. Quantitative Cell Death Analysis Using Trypan Blue

The harvested cells were stained with trypan blue to determine cell death, then the cells were observed and counted under an inverted phase-contrast microscope with 10× magnification (Zeiss, Axiovert 25, Göttingen, Germany). Cell viability was calculated by the ratio of trypan blue-positive cells to the total number of cells [34].

#### 2.5.2. Cell Morphology and Nuclear Morphological Analysis

The harvested cells were washed two times with cold PBS and fixated with 4% paraformaldehyde, then permeabilized with 0.1% Triton X-100 for 30 min. Then, they were stained with 1 µg/mL of Hoechst 33342 stain (Life Technologies, Carlsbad, CA, USA) for 20 min. Cell and nuclear morphology were detected using a Leica DMi8 confocal microscope (63× oil immersion objective) (Leica, Wetzlar, Germany), and the images were processed using ImageJ software version 2.1.4.7 [35].

#### 2.5.3. Cell Cycle Analysis

The HSF cells were harvested and rinsed in PBS, followed by fixation in cold 90% methanol for 60 min at 4 °C. They were then centrifuged at 363× *g* for 5 min at RT, and the pellet was washed with PBS and stained using PI/RNA reagent (RNase 1× PBS (100 µg/mL RNase) and PI stain (50 µg/mL)) (Cell Signaling Technology, Inc., Danvers, MA, USA), then incubated for 30 min at RT in the dark. According to Ghosh et al. [36], the cell cycle was analyzed using flow cytometry (BD Bioscience, Heidelberg, Germany).

#### 2.5.4. Evaluation of DNA Damage

The anti-genotoxicity of aqueous mushroom extracts was evaluated using an alkaline comet assay according to Sevindik et al. [2], with some procedure adjustments. After the cells were harvested, the cell pellet was re-suspended in 1× PBS. The cell suspension was mixed with melted 0.5% low-melting agarose at a ratio of 1:10 (*v*/*v*), and 30 µL of this mixture was dropped in the center of a slide pre-coated with 1% normal melting agarose and covered with a coverslip, then stored at 4 °C in the dark for 10 min. The coverslip was removed after solidification, and the slides were submerged in lysis buffer for 45 min at 4 °C in the dark. This was followed by rinsing the slide with ice-cold diH_2_O for 30 min, then immersing it in an electrophoresis tank with freshly prepared ice-cold electrophoresis buffer and incubating in the dark at 4 °C for 10 min. The electrophoresis was performed for 30 min at 26 V and 400 mA at 4 °C, then the slides were immersed two times in a neutralization buffer for 5 min at RT, followed by cold 70%, 80% and 100% ethanol for 5 min each at RT to dehydrate the slides.

Finally, the dried slides were stained with ethidium bromide (50–100 µL) on each slide. The slides were visualized under a fluorescence microscope with 400× magnification (Olympus BX41, Tokyo, Japan), and the images were taken by a DP20 digital microscope camera (Olympus, Tokyo, Japan). DNA damage was determined as a DNA damage index (DI) [37].

#### 2.5.5. Determination of ROS Level

The Reactive Oxygen Species (ROS) level was evaluated using a reactive oxygen species (ROS) ELISA Kit (Sun Long Biotech Co., Ltd., Hangzhou, China) [38]. The HSF cells were harvested, rinsed in PBS, re-suspended in 1× PBS, and then the ELISA Kit was used. The plates were analyzed by scanning an ELISA reader (Bio-Rad Benchmark, Tokyo, Japan) at O.D. 450 nm.

#### 2.5.6. RNA Isolation and Quantitative PCR

Total RNA was isolated from the HSF cells using trizol according to the manual protocol (GENEzol Reagent, Geneaid, New Taipei, Taiwan), then complementary DNA (cDNA) was synthesized using reverse transcriptase (Enzynomics, Daejeon, Republic of Korea). The products of cDNA were used directly with the SYBR Green mixture (Enzynomics, Daejeon, Republic of Korea) [39]. Primers of cytokine genes (interleukin, IL-2, IL-4, and IL-6) and telomere length genes (Telomere, 36B4) are shown in Table 1. The PCR reaction was performed at 95 °C for 10–15 min, followed by 30–45 cycles at 94 °C for 10–15 s, 60 °C for 30–60 s, and 72 °C for 15–60 s. The expression levels were determined via the 2^−ΔΔct^ method, normalized by GAPDH as an endogenous gene level [40]. The measurement of telomere lengths was calculated by the telomere/single-copy gene ratio (T/S ratio). The relative telomere ratio (T/S) = 2^−ΔΔCT^, where ΔCT = CT (telomere) − CT (36B4) [41].

### 2.6. Statistical Analysis

The data were described as means ± standard deviation (SD). Statistical analysis was performed using one-way and two-way analysis of variance (ANOVA) tests in SPSS software version 25 (SPSS Inc., Chicago, IL, USA). The *t*-test was used to evaluate the differences among the data sets. The difference between the means was considered significant at *p* < 0.05 and *p* < 0.01. The gene expression experiments were presented using the ΔΔCt method. The DNA damage index (DI) was calculated by multiplying the number of cells associated with each damage class by a conversion factor, using the formula: DI = (0 × n0) + (1 × n1) + (2 × n2) + (3 × n3) + (4 × n4) + (5 × n5), where (0 to 4) is the damage class and (n) is the number of cells in each class.

## 3. Results

### 3.1. Antioxidant Activities in Mushroom Extracts

**Figure 2:**
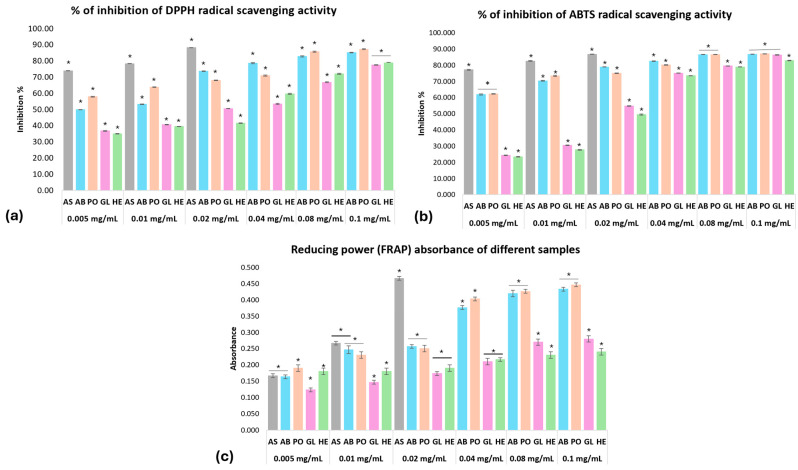
DPPH and ABTS^+^ radical scavenging activity and reducing power absorbance of different mushroom extracts. Where (**a**) DPPH^•^, (**b**) ABTS^+•^, and (**c**) reducing power (FRAP). AS (ascorbic acid) (0.005, 0.01, 0.02 mg/mL), AB (*Agaricus bisporus*), PO (*Pleurotus ostreatus*), GL (*Ganoderma lucidum*), and HE (*Hericium erinaceus*) extracts (0.005–0.1 mg/mL). Data are presented as the mean ± SD. Differences that show statistical significance at * *p* < 0.05.

DPPH^•^ and ABTS^+•^ are frequently used to evaluate antioxidants’ capability to scavenge free radicals [44]. DPPH^•^, ABTS^+•^, and FRAP demonstrate the reducing power and free radical scavenging abilities of *AB*, *PO*, *GL* and *HE* extracts presented in Figure 2a–c and Table 2. The inhibition percentages of DPPH^•^ scavenging activity for *AB*, *PO*, *GL* and *HE* extracts at 0.1 mg/mL were 85.16, 87.23, 77.45 and 78.71%, respectively. All the extracts have significant differences (*p* < 0.05) among each other, except for the *GL* and *HE* extracts, where there were no significant differences between them at 0.005, 0.01, and 0.1 mg/mL (Figure 2a). The percentage of ABTS^+•^ scavenging ability at 0.1 mg/mL was 86.78, 86.99, 86.35 and 82.94%, respectively. There were no significant differences between *AB* and *PO* extracts at 0.005, 0.08 or 0.1 mg/mL, and between *AB* and *GL* at 0.1 mg/mL (Figure 2b). Regarding the reducing power, the absorbance of the four mushroom extracts at 0.1 mg/mL was 0.43, 0.45, 0.28 and 0.24, respectively. Also, there were no significant differences between *AB* and *PO* extracts at 0.01, 0.02, 0.08 and 0.1 mg/mL, or between *GL* and *HE* at 0.02 and 0.04 mg/mL (Figure 2c).

The scavenging ability of all extracts to DPPH and ABTS^+^ radicals was significantly (*p* < 0.05) inferior (higher IC_50_ value) to that of ascorbic acid. In comparing the four extracts, the scavenging ability of *AB* and *PO* extracts to these radicals was significantly (*p* < 0.05) superior (lower IC_50_ value) to that of the other mushroom extracts. Likewise, the reducing power of all extracts was significantly (*p* < 0.05) inferior (higher EC_50_ value) to that of ascorbic acid. The *AB* and *PO* extracts showed a significantly (*p* < 0.05) superior reducing power (lower EC_50_ value) compared with *GL* and *HE* extracts (Table 2).

According to the results, all mushroom extracts could act as antioxidants and may help protect the human body from oxidative damage, which can lead to degenerative disorders and other health problems, due to their high radical scavenging activity.

### 3.2. Cell Viability by MTT Assay

**Figure 3:**
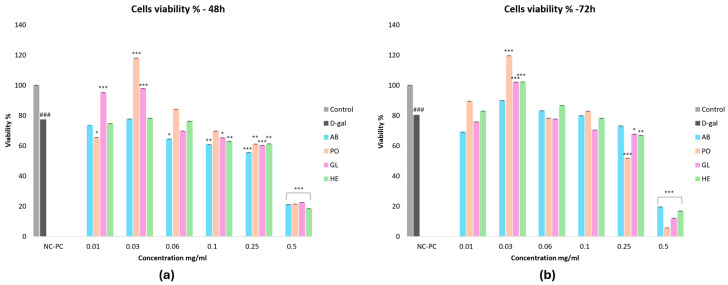
Cell viability percentage of normal HSF cells, aging cells induced by D-gal (30 g/L/48,72 h), and aging cells treated with different mushroom extracts at various doses (0.50–0.01 mg/mL) for 48 h and 72 h. (**a**) The viability after 48 h. (**b**) The viability after 72 h. Data are presented as the mean ± SD. Differences that show statistical significance at * *p* < 0.05, ** *p* < 0.01, and *** *p* < 0.001 compared with the D-gal group, and ### *p* < 0.001 compared with the control group.

The MTT assay assessed the cytoprotective effects of various mushroom extracts (0.50–0.01 mg/mL) on D-gal (30 g/L)-induced aging in HSF cells. Cells were exposed to D-gal for 72 h, followed by treatment with mushroom extracts for 48 or 72 h (Figure 3a,b). D-gal significantly reduced cell proliferation to 80.22% at 72 h, while mushroom extracts exhibited a time- and dose-dependent enhancement of cell viability on the same cells.

The optimal dose of 0.03 mg/mL of PO, GL, and HE extracts significantly enhanced cell viability (*p* < 0.001) at 72 h compared to the D-gal group. Notably, PO extracts had the greatest impact on cell survival out of all the extracts tested, followed by HE, GL, and AB, respectively. 

Table 3 summarizes the extracts’ IC_50_ values (concentration required to inhibit cell growth by 50%). PO extracts demonstrated the highest potency (IC_50_ = 0.27 ± 0.26 mg/mL) against D-gal-induced toxicity after 72 h. Based on our findings, we recommend further investigating the cytoprotective effects of these mushroom extracts using concentrations of 0.25 and 0.03 mg/mL for 72 h. 

### 3.3. Quantitative Cell Death Analysis Using Trypan Blue

**Figure 4:**
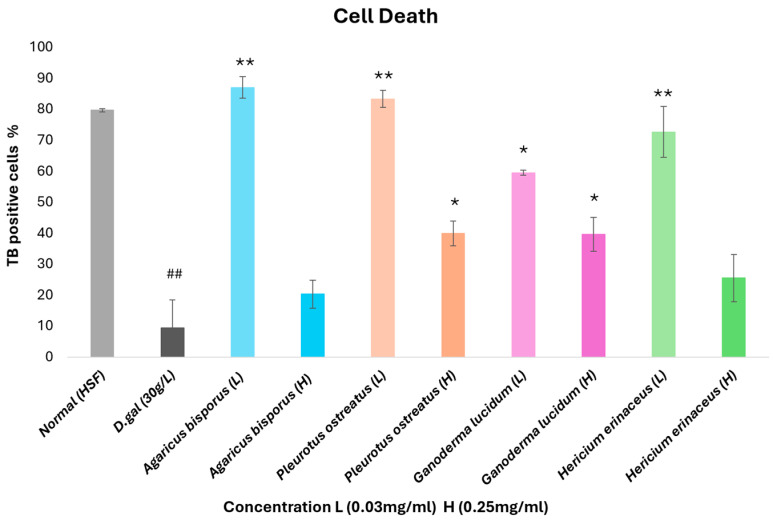
Cell death induced by D-gal and the effect of different mushroom extracts on aging cells. Trypan blue (TB) was used to stain adherent and floating cells, and the TB-positive cells were presented as a percentage. L (low concentration: 0.03 mg/mL), H (high concentration: 0.25 mg/mL). Data are presented as the mean ± SD. Differences that show statistical significance at * *p* < 0.05 and ** *p* < 0.01 compared with the D-gal group, and ## *p* < 0.01 compared with the control group.

The trypan blue test assessed necrotic and D-gal-induced cell death by counting viable cells after staining with trypan blue (TB). The percentages of viable cells relative to the total cell count were calculated and visualized in Figure 4.

Our results showed a marked increase in cell death and a significant (*p* < 0.01) decrease in proliferation in HSF cells treated with 30 g/L D-gal for 72 h compared to normal HSF cells. Interestingly, aging cells treated with a low concentration (0.03 mg/mL) of various mushroom extracts showed significantly (*p* < 0.01) enhanced proliferation compared to the effect of a high concentration (0.25 mg/mL). All mushroom extracts (0.03 mg/mL) demonstrated the potential to mitigate the aging impact of D-gal on HSF cells, with the AB group exhibiting the highest proliferation rate at 87%, followed by PO, HE, and GL, respectively.

### 3.4. Cell and Nuclear Morphological Analysis 

**Figure 5:**
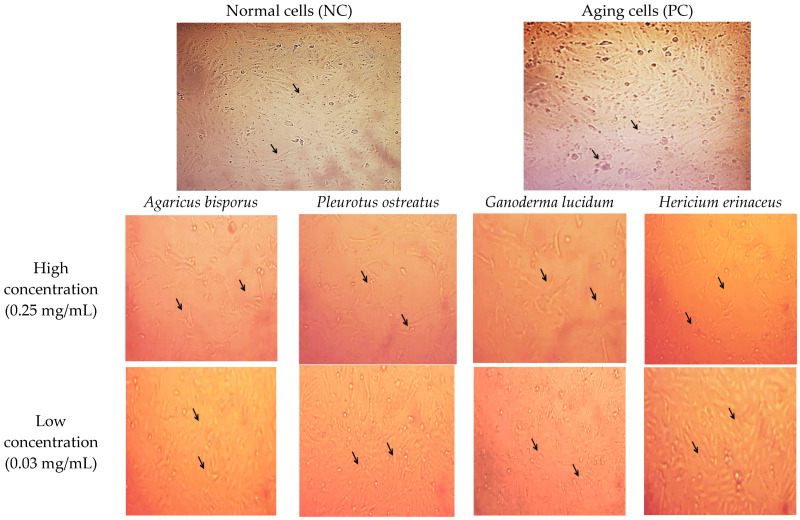
Morphological cell changes of normal HSF cells and aged HSF cells induced by D-gal 30 g/L/72 h and after being treated with different mushroom extracts at various concentrations (0.25–0.03 mg/mL/72 h), under the inverted phase-contrast microscope at 5× magnification, the changes are illustrated by arrows. The normal cells were observed to grow normally with a spindle shape and even distribution, while aging cells showed abnormal behavior with a wider shape, increased space between the cells, and increased senescent cells. The cells treated with the high concentration (0.25 mg/mL/72 h) expressed abnormal shapes and fewer cells, but the cells treated with the low concentration (0.03 mg/mL/72 h) showed normal growth with normal shape and even distribution.

**Figure 6:**
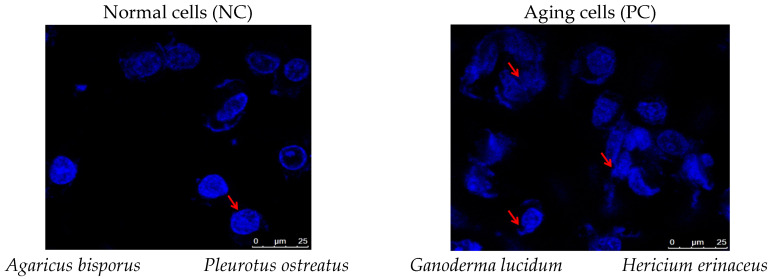

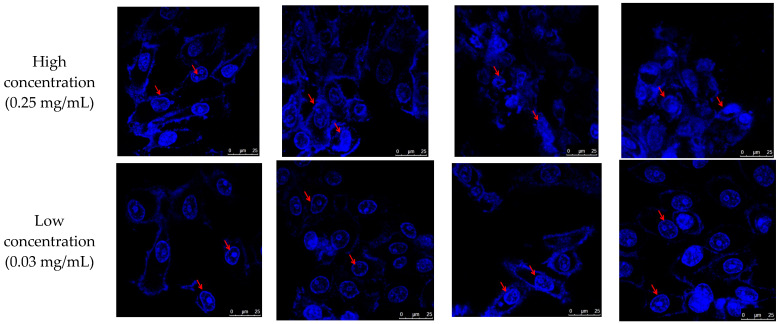
Nuclear morphology of normal HSF cells, aged HSF cells induced by D-gal 30 g/L/72 h, and after treatment with different mushroom extracts at various concentrations (0.25–0.03 mg/mL/72 h). Examined under a confocal microscope at 63× magnification after being stained with Hoechst 33342 stain, the changes are illustrated by arrows. The normal HSF cells showed normal nucleus shape, while the aging cells observed abnormal shapes of the nucleus and cells, a shrunken-shaped nucleus, DNA fragmentation, and chromatin clumping. The aging cells treated with the high concentration (0.25 mg/mL/72 h) did not show significant improvement, but the aging cells treated with low concentration (0.03 mg/mL/72 h) showed normal morphology of nuclei with round, regular shapes.

Using inverted and confocal microscopy, we examined the morphological effects of D-gal on cells and nuclei. Mushroom extracts were tested for their ability to mitigate these changes, providing insights into their potential anti-aging and protective benefits (Figure 5 and Figure 6).

Under the inverted microscope, aging cells displayed abnormal shapes, decreased proliferation, increased senescence, and increased space between the cells compared to normal cells (arrows). However, treatment with mushroom extracts, especially at a low concentration (0.03 mg/mL), mitigated these effects and restored normal cell morphology and distribution (arrows) (Figure 5).

Nuclear morphology was estimated using Hoechst 33342 staining and confocal microscopy (Figure 6). D-gal-induced aging cells displayed characteristic apoptotic features, including DNA fragmentation, shrunken nuclei, and chromatin clumping (arrows). Conversely, mushroom extracts at a low concentration (0.03 mg/mL) significantly reduced apoptotic markers and restored normal nuclear shape (arrows) after 72 h, unlike the high concentration (0.25 mg/mL). According to our research, all four mushroom extracts possess anti-aging potential; however, PO and HE extracts showed the greatest improvements in cell morphology and decreased apoptosis.

### 3.5. Cell Cycle Analysis

**Figure 7:**
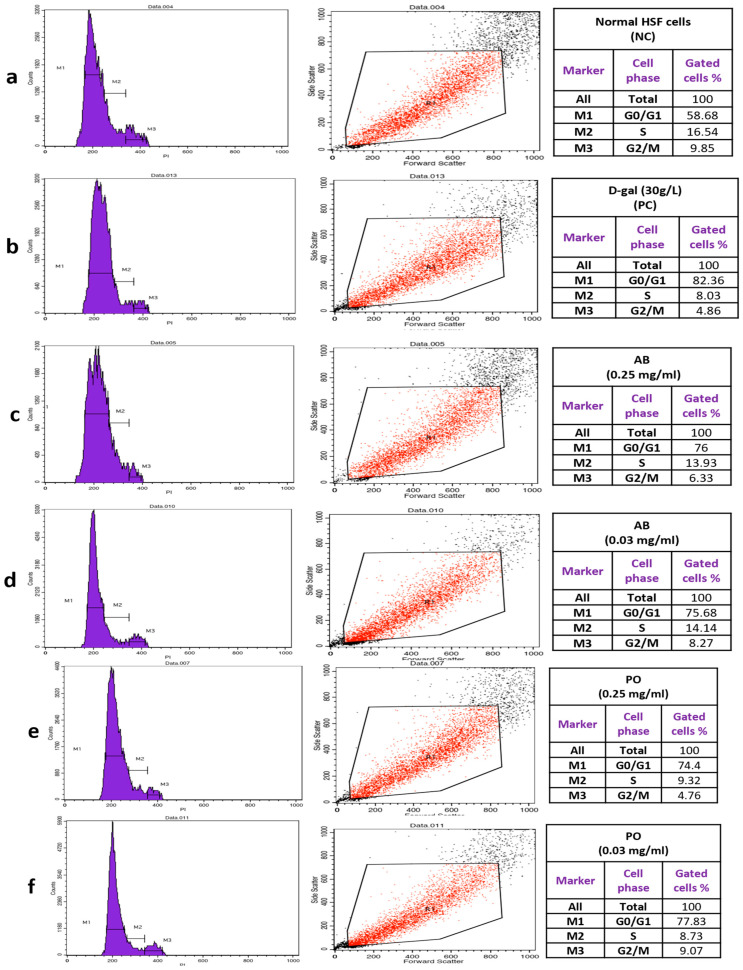

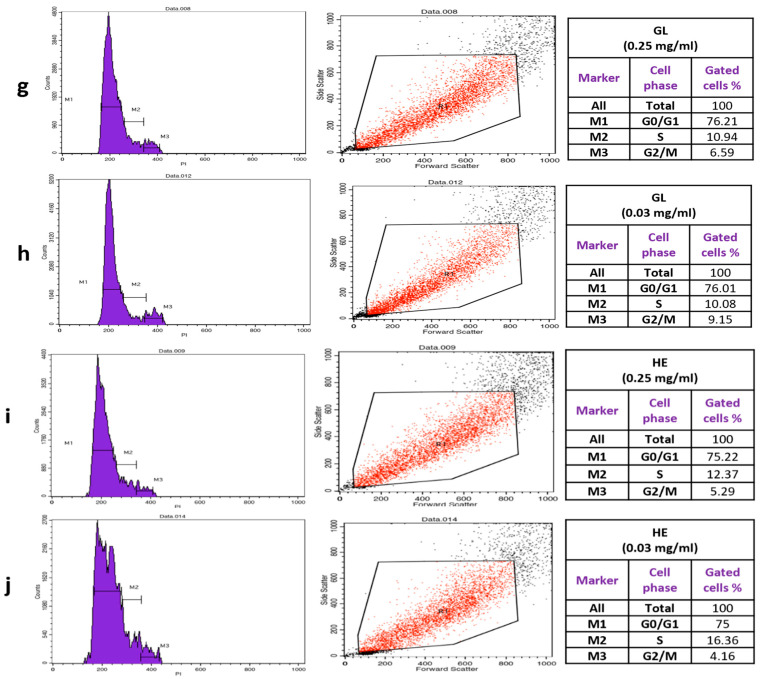
Cell cycle distribution of normal HSF cells and aging cells induced by D-gal (30 g/L/72 h) and aging cells treated with different mushroom extracts. (**a**): Normal HSF cells, NC (Negative control), (**b**): D-gal group, PC (Positive control), (**c**): AB (*Agaricus bisporus*) (0.25 mg/mL/72 h), (**d**): AB (0.03mg/mL/72 h), (**e**): PO (*Pleurotus ostreatus*) (0.25 mg/mL/72 h), (**f**): PO (0.03 mg/mL/72 h), (**g**): GL (*Ganoderma lucidum*) (0.25 mg/mL/72 h), (**h**): GL (0.03 mg/mL/72 h), (**i**): HE (*Hericium erinaceus*) (0.25 mg/mL/72 h), (**j**): HE (0.03 mg/mL/72 h). The aging HSF cells showed an increase of arrested cells at the G0/G1 phase by 82.36%, while after being treated with mushroom extracts exhibited a decrease in cell arrest at the G0/G1 phase with approximately 75% gated cells and enhanced the synthesis of DNA by increasing the cells at the S phase where the highest percentage was 16.36% in the HE extract (0.03 mg/mL).

The cell cycle distribution of HSF cells was examined using flow cytometry and PI staining technique (Figure 7a–j). Normal HSF cells exhibited a predominantly G0/G1 cell cycle distribution, with over 50% of cells in this phase and more than 15% in the S phase (Figure 7a). After 72 h of D-gal exposure, HSF cells were arrested in the G0/G1 phase at 82.36%, while S phase cells decreased to 8.033%. This indicates that D-gal inhibits cell division and arrests the cell cycle (Figure 7b).

In contrast, treatment with mushroom extracts at concentrations of 0.03 and 0.25 mg/mL reduced cell cycle arrest in the G0/G1 phase to 75%. Additionally, these extracts promoted the transition from G0/G1 to the S phase, with the highest percentage of cells in the S phase observed with the HE extracts at 0.03 mg/mL (16.36%). This suggests that mushroom extracts enhance DNA synthesis (Figure 7j).

### 3.6. Comet Assay (Single-Cell Gel Electrophoresis)

**Figure 8:**
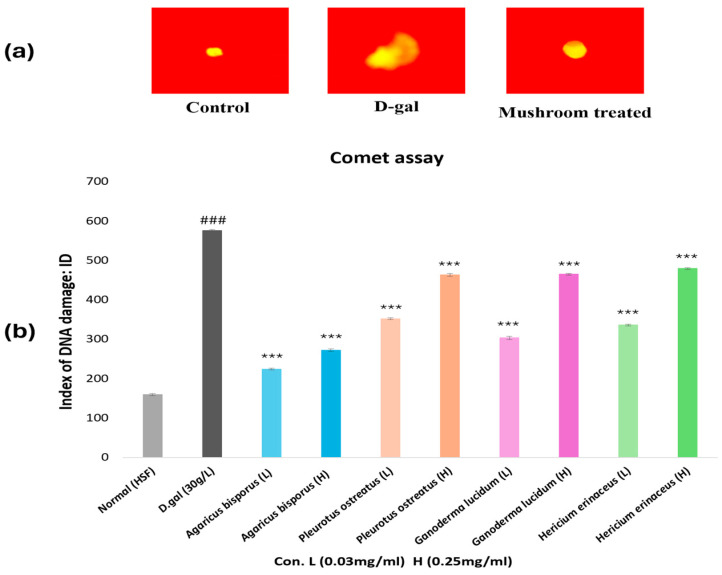
Comet assay analysis of aging cells induced by D-gal (30 g/L/72 h) and aging HSF cells treated with mushroom extracts, where L: (0.03 mg/mL/72 h), H: (0.25 mg/mL/72 h). (**a**): The HSF cell’s shape under the fluorescence microscope after staining with ethidium bromide presented the different types of DNA damage, (**b**): Chart of the DNA-damaged index. The data are presented as the mean ± SD. Differences that show statistical significance at *** *p* < 0.001 compared with the D-gal group, ### *p* < 0.001 compared with the control group.

An alkaline Comet assay was performed to assess the protective effects of mushroom extracts against D-gal-induced DNA damage (Figure 8a,b). Our results demonstrated a significant (*p* < 0.001) increase in DNA damage (comet tail) in HSF cells exposed to D-gal compared to the control group (comet head), confirming its genotoxic effects. All mushroom extracts (AB, PO, GL, and HE) significantly (*p* < 0.001) mitigated D-gal-induced DNA damage in HSF cells at both 0.25 and 0.03 mg/mL concentrations. AB extract was the most potent in reducing DNA damage, particularly at the 0.03 mg/mL concentration. These findings suggest that mushroom extracts, especially AB, may have potential anti-aging properties and could protect against oxidative DNA damage.

### 3.7. Determination of Reactive Oxygen Species (ROS) Level

**Figure 9:**
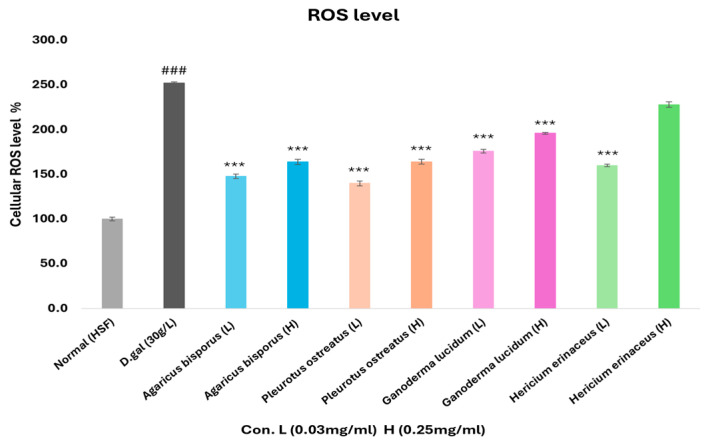
Reactive oxygen species (ROS) levels of aging HSF cells induced by D-gal (30g/L/72 h), and after being treated with different mushroom extracts (0.03 and 0.25 mg/mL) for 72 h. It was determined by ELISA assay. The data are presented as the mean ± SD. Differences that show statistical significance at *** *p* < 0.001 compared with the D-gal group, ### *p* < 0.001 compared with the control group.

The level of ROS was analyzed to identify oxidative stress in aging HSF cells after being exposed to 30 g/L D-gal for 72 h, and the antioxidant effect of different mushroom extracts (Figure 9).

Aging HSF cells showed a significant increase (*p* < 0.001) in intracellular ROS level by 252% compared with normal HSF cells, indicating that D-gal induced oxidative stress in HSF cells by promoting the accumulation of intracellular ROS. As predicted, the groups that received mushroom extracts (0.03 and 0.25 mg/mL) for 72 h treatment showed a significant reduction (*p* < 0.001) in ROS levels compared with the D-gal group. The mushroom extracts effectively reduced harmful ROS levels and protected the cells from oxidative damage caused by D-gal.

### 3.8. Molecular Analysis (qRT-PCR)

#### 3.8.1. Quantitative Real-Time PCR Analysis of Immunomodulatory and Inflammation-Regulation Genes

**Figure 10:**
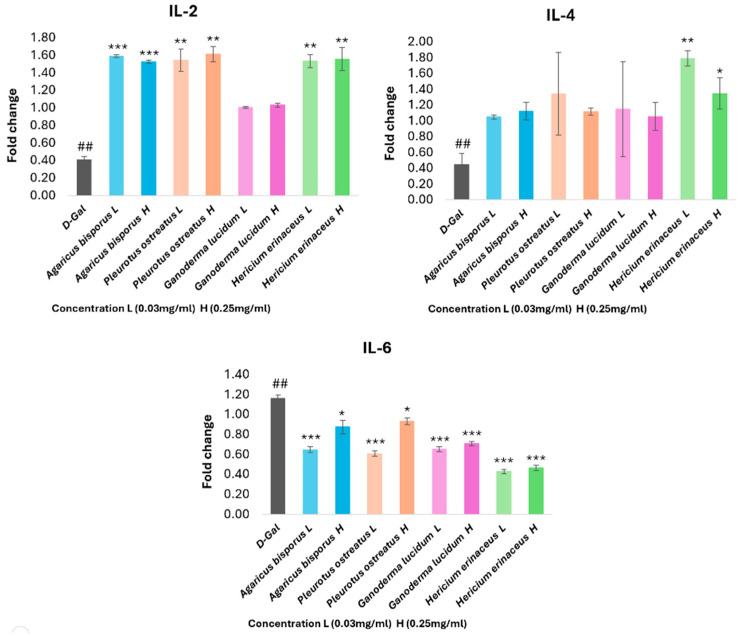
Influence of four mushroom extracts on the levels of cytokine mRNA expression of IL-2, IL-4, and IL-6 in the aging HSF cells induced by D-gal (30 g/L/72 h) and after being treated with different mushroom extracts (0.03–0.025 mg/mL/72 h). The data are presented as the mean ± SD. Differences that show statistical significance at * *p* < 0.05, ** *p* < 0.01, and *** *p* < 0.001 compared with the D-gal group, and ## *p* < 0.01 compared with the control group.

To investigate the impact of aging on immune function, we quantified the gene expression of key cytokines involved in immunomodulation and inflammation. Using qRT-PCR, we measured the levels of interleukin-2 (IL-2), interleukin-4 (IL-4), and interleukin-6 (IL-6) mRNA (Figure 10).

The IL-2 and IL-4 genes showed a significant decrease (*p* < 0.01) in the D-gal group (aging model) compared with the control group. Furthermore, IL-2 expression increased significantly (*p* < 0.01) (*p* < 0.001) in all mushroom extract groups, except for the GL group. There were no significant differences between low- and high-concentration groups. IL-4 expression was significantly upregulated in the HE group (*p* < 0.01). Specifically, the low-concentration (0.03 mg/mL) group exhibited an increase in IL-4 expression compared to the high-concentration (0.25 mg/mL) group. The expression of pro-inflammatory cytokine IL-6 genes significantly increased (*p* < 0.01) in the D-gal group, but all aging cell groups treated with mushroom extracts showed a significant decline (*p* < 0.05, *p* < 0.001) in gene expression, with no significant differences in expression levels between the concentrations, except the AB and PO groups, where the low concentration showed lower expression than the high concentration. These results suggest that the mushroom extracts might stimulate an immune response and reduce inflammation. 

#### 3.8.2. Real-Time PCR Analysis of Telomere Length Genes

The telomere length of HSF cells was measured to examine the impact of D-gal on telomere length and the potential anti-aging properties of various mushroom extracts. The aging cells induced by D-gal (30 g/L/72 h) showed a significant decrease (*p* < 0.05) in gene expression of telomere length compared with the normal cells.

**Figure 11:**
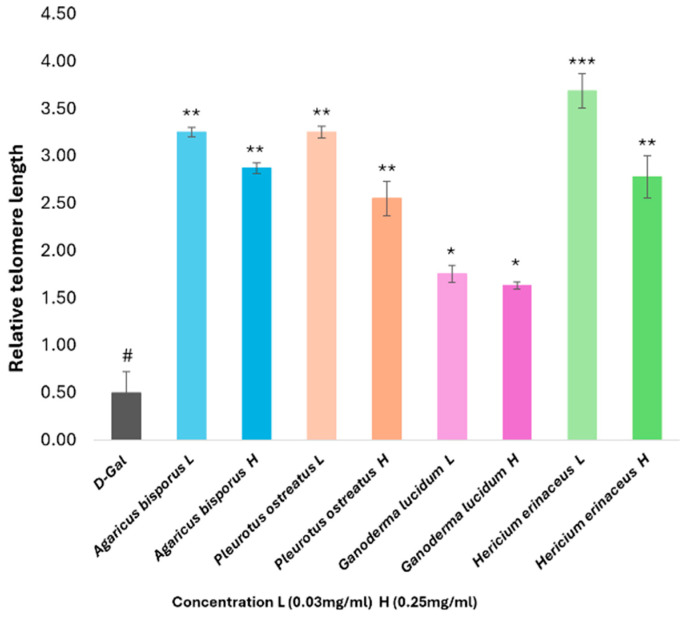
Influence of four mushroom extracts on the relative telomere length of aging HSF cells induced by D-gal (30 g/L/72 h) after being treated with different mushroom extracts (0.03–0.25 mg/mL/72 h). The data are presented as the mean ± SD. Differences that show statistical significance at * *p* < 0.05, ** *p* < 0.01, and *** *p* < 0.001 compared with the D-gal group, #*p* < 0.05 compared with the control group.

In contrast to the D-gal group, treatment with mushroom extracts significantly increased telomere length. Among the mushroom extracts tested, the HE group with low concentration (0.03 mg/mL) demonstrated the most effective protection against D-gal on telomere shortening (*p* < 0.001), followed by the PO, AB, and GL groups, respectively (Figure 11).

## 4. Discussion

The aging process is strongly associated with oxidative stress as it alters the structure and function of the body’s tissues and organs, as well as the structures of micro-molecules [45]. Adding natural antioxidant supplements to meals is an easy and efficient way to manage oxidative stress [46]. While previous studies have explored the antioxidant properties of certain mushroom species, our research differs by providing a comprehensive evaluation of both antioxidant and anti-aging effects on aging cells. Additionally, we included [specific mushroom species] in our study, which have not been extensively investigated in this context. 

In this study, we investigated the antioxidant activity of four different types of edible and medicinal mushroom extracts (*Ganoderma lucidum*, *Hericium erinaceus*, *Pleurotus ostreatus*, and *Agaricus bisporus*) by measuring the inhibition level of DPPH^•^, ABTS^+•^, and the reducing power. All extracts showed high antioxidant activity, particularly at a concentration of 0.1 mg/mL. *Pleurotus ostreatus* extract showed the highest scavenging activity (the lowest IC_50_) for DPPH^•^ and ABTS^+•^ radicals and the strongest reducing power (the lowest EC_50_) compared to the other mushroom extracts. Recent studies show that *Pleurotus ostreatus* has a significant DPPH^•^ scavenging rate of 66.12% [47] and 71.29% in ethanolic extracts, according to Dundar et al. [48]. *Agaricus bisporus* polysaccharides exhibit potent DPPH^•^ scavenging properties with a concentration-dependent effect [49]. *A. bisporus* and *P. ostreatus* extracts inhibit DPPH^•^ by 86.33% and 43.88%, respectively [50]. Aqueous extracts of *A. bisporus* and *P. ostreatus* show the highest DPPH^•^ scavenging capacity [51]. Several studies found that *P. ostreatus* and *A. bisporus* extracts have strong reducing power (2.81 and 2.31, respectively) [51]. *A. bisporus* polysaccharides showed improved antioxidant properties and reduced power in mushrooms [52]. This study investigated the antioxidant and anti-aging potentials of various mushrooms in aging HSF cells induced by D-galactose at a concentration of 30 g/L for 72 h; this concentration causes cell aging according to previous research [32,53]. Results show the highest viability was at 0.03 mg/mL for 72 h for all mushroom extracts compared with the D-gal group. The lowest IC_50_ value was 0.24 ± 0.04 mg/mL for *Agaricus bisporus* extract at 48 h and 0.27 ± 0.26 mg/mL for *Pleurotus ostreatus* extract at 72 h. According to several studies, D-gal caused oxidative stress and significantly reduced the cell viability of the different types of cells [54]. Furthermore, our result agrees with the finding of Nitthikan et al., where the cell viability of brown *A. bisporus* aqueous extract was 97.86% at 0.03 mg/mL of HaCaT cells, which was the highest viability compared to the other concentrations [55]. The peptide fractions of *A. bisporus* can also protect against the oxidative stress of H_2_O_2_ in cells by inducing cell viability [56]. According to Choi et al. [57], aqueous extracts of *P. ostreatus* and *H. erinaceus* increased the cell viability of aged human dermal fibroblast cells (HDFs). Also, the polysaccharide fractions of *P. ostreatus* induced cell viability [58]. Other studies showed the preventive effect of *H. erinaceus* against oxidative stress and aging in cells by increasing the rate of cell viability [59,60,61]. *G. lucidum* extracts showed a protective effect against oxidative stress in cells by inducing cell viability [62,63]. In addition, cell death assessed by the trypan blue test confirmed the cell viability results, with the proliferation rate significantly decreased in aging HSF cells, as confirmed by Li et al. [34]. In contrast, the treated cells of all mushroom extracts at the low concentration (0.03 mg/mL) showed a high proliferation rate; the PO, AB, and HE groups had the highest proliferation rate, followed by the GL group, compared to the D-gal group. The studied mushroom extracts have shown potential benefits in protecting cells from oxidative stress and promoting cell viability and proliferation.

Cell shape is one of the morphological characteristics of senescent cells, with a wide shape [64]. Therefore, we also examined the cell morphology and nuclear morphology of the senescent HSF and senescent HSF-treated cells. Under the inverted and confocal microscope, it was observed that the aging HSF cells had abnormal shapes with a wider shape and low proliferation rate, more senescent cells with abnormal shapes of the nucleus, shrunken shaped nucleus, DNA fragmentation, and chromatin clumping, which is a morphological sign of apoptosis. There have been previous reports that D-gal caused a reduction in the proliferation rate and viability of adult dermal fibroblast (ADF) cells [32] and N2a neuroblastoma cells [34]. They also showed abnormal cell morphology, reduced nerve filaments, cell clustering, and chromatin condensation in PC12 cells (Alzheimer’s model cells) [35]. In contrast, the aging cells treated with the low concentration (0.03 mg/mL/72 h) of mushroom extracts reflected the effect of D-gal, which showed normal growth with normal shape, even distribution, and normal morphology of nuclei compared to the cells treated with the high concentration (0.25 mg/mL/72 h). PO and HE extracts showed the best improvement in nuclei and cell morphology. According to previous studies, the polysaccharides of *P. ostreatus* were observed to reverse the aging effect of H_2_O_2_ on PC12 and C2C12 cells [65,66]. *G. lucidum* polysaccharides (GLPs) also protected HSF cells from H_2_O_2_ oxidative stress by improving the morphological state of the cells [63] and preventing neuritis after exposure to Aβ25–35 [67]. *H. erinaceus* significantly reduced apoptotic cells in IPEC-J2 stressed cells induced by H_2_O_2_ [59]. They inhibited the apoptosis of stressed IPEC-J2 cells induced by the Fusarium toxin deoxynivalenol [68]. This suggests that the restorative effect of the mushroom extract counteracts the necrotic effect of D-gal in HSF cells. 

DNA damage and reduced cell growth are caused by severe oxidative stress, which can be observed by cell cycle analysis using flow cytometry. One of the primary markers of cellular senescence is usually considered to be a sub-G1 peak [66]. In our results, the cell cycle distribution showed an increase in arrested cells in the G0/G1 phase with 82.36% in aged HSF cells compared to normal cells, indicating the effect of D-gal on cell proliferation and cell cycle arrest. In contrast, the treated groups showed a decrease in arrested cells in the G0/G1 phase and an increase in cells in the S phase, with the HE extracts (0.03 mg/mL) being the best at 16.36% in the S phase, which enhanced DNA synthesis and promoted the start of cell division. This was consistent with previous research, where D-gal-induced senescent cells showed an increase in arrested cells in the G1 phase [38,54]. On the other hand, the polysaccharides of *P. ostreatus* led to a decrease in cells in the G0/G1 phase in aging PC12 and C2C12 cells [65,66].

According to the results of the comet assay, the aging HSF cells showed a significant increase (*p* < 0.001) in the DNA-damaged cells index, which inferred the cytotoxic effect of D-gal, consistent with previous studies [69,70]. In contrast, a significant decrease (*p* < 0.001) in DNA-damaged cells was observed in all treated groups, with AB extract being the most protective extract, followed by GL, HE, and PO extracts at 0.03 mg/mL. A previous study demonstrated the anti-aging effects of *G. leucocontextum* against the oxidative effect of D-gal by decreasing DNA damage levels [70]. The ethanolic and aqueous extracts of *G. lucidum* also prevented H_2_O_2_-induced DNA damage [71]. According to previous studies, *A. bisporus* extracts have an antigenotoxic effect by reducing DNA damage caused by oxidative stress [72,73]. The ethanolic extract of *H. erinaceus* was observed to have a protective effect on genotoxicity against DNA damage induced by ethyl methanesulfonate (EMS) [74]. This suggests that these mushroom extracts may serve as a natural prophylactic agent against DNA damage induced by D-gal.

To study oxidative stress and its association with aging, and to evaluate the antioxidant potential effects of various mushroom extracts, the ROS levels were assessed. The overproduction of ROS damages cells and promotes aging [75]. Our results showed a significant increase (*p* < 0.001) in ROS production by a ratio of 252% in aging HSF cells, inducing early cellular senescence. Also, various studies have reported that D-gal induces an increase in ROS levels in cells and stimulates oxidative stress and aging [38,53,54,61]. On the other hand, all treated groups (0.03 mg/mL) observed a significant decrease in ROS level, where the PO extract showed the most reduction. According to Feng et al. [76], the level of ROS was increased in cells induced by D-gal, whereas it decreased after being treated with *A. blazei*. The peptide fractions of *A. bisporus* also reduced the increase of ROS levels induced by H_2_O_2_ [56], and treatment with polysaccharides from *P. ostreatus* prevented the oxidative effect [58,65] by reducing ROS levels. The aqueous extract of *G. lucidum* was observed to reduce ROS levels in aging and stressed cells [62,63]. According to various research studies, *H. erinaceus* prevents oxidative stress in cells by reducing ROS levels of aging cells [59,60,68].

Regarding the gene expression study, our results showed a significant decrease (*p* < 0.01) in the gene expression of IL-2 and IL-4 and an increase in the gene expression level of IL-6 in the D-gal senescent cells, indicating the effect of D-gal on immunological function and inflammation in HSF cells, where the increase in inflammatory activity (IL-6) is associated with aging [77]. This is consistent with previous studies where the level of IL-6 in senescent human astrocytic crT cells was increased by D-gal [78]. It is also associated with Alzheimer’s disease [79] and Parkinson’s disease [80]. Similarly, the cytokines IL-2 and IL-4 are associated with aging and are reduced in PD patients [81].

Otherwise, AB, PO, and HE extracts showed a significant increase in IL-2 gene expression, with no significant differences between the concentrations, while the GL extract did not show a significant effect. The HE extract is the only one that showed a significant increase in the IL-4 gene. All mushroom extracts also caused a downregulation of IL-6 gene expression level in aging HSF cells, with the HE group being most effective at a concentration of 0.03 mg/mL/72 h. That indicates all mushroom extracts can enhance immunological function and decrease inflammation in the aging cell model. Similar results have been documented, with the aqueous extract of *A. bisporus* [55] and its methanolic extracts [82] reducing inflammation by decreasing the level of IL-6 in inflamed cells. In addition, the polysaccharide fractions of *P. ostreatus* observed an anti-inflammatory effect against aging cells by decreasing the level of IL-6 [58], while in the study by Moro et al. [82] the methanolic extracts of *P. ostreatus* did not show significant results. According to Xu et al. [83], *G. lucidum* polysaccharides (GLP) could reduce the level of IL-6 in insulin model mice and increase the level of IL-2 in human PBMC cells, while low levels of IL-4 were observed [84]. Davis et al. also observed that aqueous and solid extracts of *G. lucidum* and *H. erinaceus* can enhance immune activity (IL-2, IL-4) and reduce cellular inflammation (IL-6) [85]. According to Wu et al. [86], *H. erinaceus* has immunomodulatory potential and has been observed to promote the secretion of IL-2 and IL-4 and has immunoregulatory activity by regulating the secretion of IL-6.

Telomere length was significantly decreased (*p* < 0.001) in D-gal-induced aging HSF cells. Previous studies showed the effect of D-gal on telomere length in cells, which was shorter in aging cells than in normal cells [38]. In our experiment, the aging cells treated with the different mushroom extracts showed opposite results, with the HE extract showing higher expression at a low concentration (0.03 mg/mL). This indicates the protective effect of all mushroom extracts against D-gal on telomere length. According to Choi et al. [57], the aqueous extracts of *P. ostreatus* and *H. erinaceus* could be an effective anti-aging therapeutic in the future. These findings imply that all mushroom extracts can enhance antioxidant status throughout aging and reduce the incidence of age-related diseases linked to free radicals. While this study provides preliminary evidence for all mushroom species extracts, further research is needed to focus on the chemical characterization of the extract using techniques such as [HPLC, GC-MS, etc.] to identify the bioactive compounds responsible for the observed effects. This information would be valuable for developing standardized formulations and for understanding the mechanism of action.

## 5. Conclusions

In conclusion, our study provides novel evidence for the ability of mushroom extracts to promote cell proliferation and reduce DNA damage in aging cells. These findings are particularly significant because they suggest a potential therapeutic application for age-related diseases, where the studied species of mushrooms (*Ganoderma lucidum*, *Hericium erinaceus*, *Pleurotus ostreatus*, and *Agaricus bisporus*) showed antioxidant, anti-aging, immunomodulatory, and anti-inflammatory potential. This potential makes them a promising alternative to artificial antioxidants. The studied mushroom extracts exhibited the ability to restore cell viability, increase cell proliferation, improve DNA synthesis, prevent telomere shortening, act as a natural prophylactic agent against D-gal-induced DNA damage, and reduce oxidative stress in aging cells by reducing ROS levels. This implies their power to enhance overall antioxidant status and reduce the risk of age-related illness. Therefore, our current data demonstrate the efficacy of a nonpharmacological strategy based on dietary supplementation with different mushroom extracts. These findings contribute to the growing field of bio-based research and One Health applications for societal improvement, offering a sustainable and natural solution for improving public health and decreasing agricultural waste. Further research is needed to fully elucidate the mechanisms of action and optimize the use of mushroom-derived compounds for therapeutic purposes.

## Figures and Tables

**Table 1:** RT-PCR primer design sequences.

Primers	Forward Primer	Reverse Primer	Ref.
IL-2	GGA AAC ACA GGA ACA ACT GGA	TTC AAT TCT GTG ACC TTC TTG G	[42]
IL-4	AGA GCT CGG TGA CCT CAG AC	CTT GCA TGG CGG TCT TTA G	
IL-6	GAA AAC ACC AGG GTC AGC AT	CAG CCA CTG GTT TTT CTG CT	
Telomere	CGG TTT GTT TGG GTT TGG GTT TGG GTT TGG GTT TGG GTT	GGC TTG CCT TAC CCT TAC CCT TAC CCT TAC CCT TAC CCT	[43]
36B4-R	ACT GGT CTA GGA CCC GAG AAG	TCA ATG GTG CCT CTG GA G ATT	
GAPDH	ATC AAG TGG GGT GAT GCT GGT	CCT GCT TCA CCA CCT TCT TGA	[39]

**Table 2:** IC_50_ and EC_50_ values of four types of mushroom extracts.

Samples	DPPH^•^ (IC_50_) (mg/mL)	ABTS^+•^ (IC_50_) (mg/mL)	Reducing Power (FRAP) (EC_50_) (mg /mL)
Ascorbic acid	0.003 ± 0.01 ^a^	0.003 ± 0.01 ^a^	0.021 ± 0.02 ^a^
*Agaricus bisporus*	0.005 ± 0.01 ^c^	0.004 ± 0.02 ^b^	0.116 ± 0.04 ^c^
*Pleurotus ostreatus*	0.004 ± 0.01 ^b^	0.004 ± 0.01 ^b^	0.111 ± 0.03 ^b^
*Ganoderma lucidum*	0.020 ± 0.03 ^d^	0.018 ± 0.05 ^c^	0.181 ± 0.01 ^d^
*Hericium erinaceus*	0.024 ± 0.02 ^e^	0.020 ± 0.03 ^d^	0.211 ± 0.05 ^e^

Reported values are the mean ± SD of three replicates. Means in the same column followed by different lowercase letters are significantly different (*p* < 0.05). IC_50_ (mg/mL): inhibitory concentrations at which 50% of DPPH-ABTS^+^ radicals are scavenged. EC_50_ (mg/mL): effective concentration at which the absorbance is 0.5.

**Table 3:** IC_50_ values of aging cell viability after 48 and 72 h of treatment with mushroom extracts.

Extracts	(IC_50_) (48 h) (mg/mL)	(IC_50_) (72 h) (mg/mL)
*Agaricus bisporus* (AB)	0.24 ± 0.04	0.32 ± 0.18
*Pleurotus ostreatus* (PO)	0.31 ± 0.06	0.27 ± 0.26
*Ganoderma lucidum* (GL)	0.29 ± 0.05	0.29 ± 0.18
*Hericium erinaceus* (HE)	0.27 ± 0.04	0.31 ± 0.20

Data are presented as the mean ± SD. IC_50_: 50% decrease in cell inhibition.

## Data Availability

The original contributions presented in the study are included in the article, further inquiries can be directed to the corresponding author.
